# Amazonian Fungal Diversity and the Potential of Basidiomycetes as Sources of Novel Antimicrobials

**DOI:** 10.3390/biology15030261

**Published:** 2026-01-31

**Authors:** Luana C. R. M. dos Santos, Juan D. R. de Almeida, Naira S. O. de Sousa, Flávia da S. Fernandes, João F. V. Ennes, Hagen Frickmann, João V. B. de Souza, Érica S. de Souza

**Affiliations:** 1 Multicenter Program in Biochemistry and Molecular Biology, School of Health Sciences (ESA), Amazonas State University (UEA), Av. Carvalho Leal, 1777, Cachoeirinha, Manaus 69065-001, AM, Brazil; lcrms.dbm23@uea.edu.br (L.C.R.M.d.S.); jdra.dbm@uea.edu.br (J.D.R.d.A.); esdsouza@uea.edu.br (É.S.d.S.); 2 Mycology Laboratory, Coordination of Society, Environment and Health, National Institute for Amazonian Research (INPA), Av. André Araújo, 2936, Aleixo, Manaus 69067-375, AM, Brazil; nsods.dbb21@uea.edu.br (N.S.O.d.S.); flaviafernandes19@gmail.com (F.d.S.F.); jfbiomedic@hotmail.com (J.F.V.E.); 3 Department of Medical Microbiology, Virology and Hygiene, University Medicine Rostock, 18057 Rostock, Germany; 4 Department of Microbiology and Hospital Hygiene, Bundeswehr Hospital Hamburg, 22049 Hamburg, Germany

**Keywords:** Amazon, fungal diversity, basidiomycetes, bioprospecting, secondary metabolic pathways

## Abstract

The Amazon Forest harbors one of the largest fungal diversities on the planet, occupying a wide variety of ecological niches comprising *terra firme* (non-flooded forest), *várzea* (white-water floodplains), and *igapó* (black-water floodplains). In this review article, we examine Amazonian fungal diversity based on three complementary approaches—culture-based surveys, in situ inventories of macrofungi, and environmental DNA/metagenomic analyses—discussing advances, limitations, and contributions to regional mycological knowledge. Subsequently, we present a critical synthesis of the potential of Amazonian basidiomycetes regarding the production of metabolites with antimicrobial activity, highlighting the main genera reported in the literature, the chemical classes involved (e.g., terpenes, steroids, quinones, and bioactive peptides), and the metabolic pathways responsible for their biosynthesis. The integration between biodiversity and bioprospecting underscores the importance of Amazonian fungi both for understanding ecological processes and for the development of new solutions to the antimicrobial resistance challenge. This work seeks to fill current gaps in the academic literature and to contribute to future strategies for the conservation and sustainable use of regional mycobiota.

## Simple Summary

The Amazon rainforest harbors an extraordinary diversity of fungi that helps to sustain forest processes such as decomposition, nutrient cycling, and ecological interactions with plants and animals. At the same time, the world is facing a growing problem of infections that are difficult to treat because microorganisms become resistant against existing antibiotics and antifungal drugs. This review brings together what is currently known about fungal diversity across major Amazon ecosystems and highlights basidiomycetes as promising sources of natural molecules with antimicrobial activity. We summarize how these fungi are detected using traditional culture methods and modern molecular approaches, which types of bioactive compounds have been reported, and which genera have shown the most promising potential as producers of anti-microbially active drugs. We also discuss key gaps that limit the research progress in this field, including uneven sampling across geographic regions, technical challenges in cultivating many species, and the need to link compounds to their biosynthetic pathways and ecological roles. By mapping current evidence and priorities, this review aims to support more targeted bioprospecting efforts and to encourage approaches that protect biodiversity while enabling new strategies for antimicrobial discovery.

## 1. Introduction

Antimicrobial resistance (AMR) is one of the most important challenges to global public health in the 21st century. It is estimated that infections caused by resistant microorganisms are responsible for more than 1.27 million deaths annually, with projections indicating that this number may exceed 10 million per year by 2050 if effective measures are not implemented [1,2]. The emergence of bacteria which are resistant to multiple classes of antibiotics directly undermines the efficacy of essential medical interventions, such as surgery, chemotherapy, and intensive care. The slowdown in the development of new antibiotics, combined with partly excessive and indiscriminate use of existing drugs, accelerates this process, jeopardizing decades of progress in modern medicine [3,4]. In view of this challenging scenario, the search for new antimicrobial molecules has become an international priority, leading institutions such as the World Health Organization (WHO) to establish lists of priority pathogens and to encourage research into alternative sources of antibiotics, particularly those based on underexplored natural resources [5].

Historically, fungi have played a central role in the discovery of antimicrobial agents. Penicillin, derived from *Penicillium notatum*, revolutionized medicine in the 20th century and is internationally considered the starting point of the antibiotic era. Since then, several other compounds with antibacterial, antifungal, antiviral, and antiparasitic activities have been isolated from fungi, including cephalosporins, griseofulvin, and echinocandins [6]. Basidiomycetes, in particular, have emerged as a still underexplored yet highly promising source of bioactive substances with complex and innovative chemical structures [6,7,8]. Macroscopic fungi such as the genera *Ganoderma*, *Trametes*, and *Cortinarius* have demonstrated the capacity of producing secondary metabolites with potent antimicrobial activity, frequently associated with specific ecological traits, including wood decomposition, microbial competition in soil, and chemical defense against predators [9,10,11,12]. Moreover, the metabolic diversity of fungi is amplified by their complex biosynthetic routes involving polyketides, terpenes, alkaloids, steroids, and non-ribosomal peptides, many of which have yet to be characterized for their bioactivity [13].

The Amazon Rainforest is recognized as a major global hotspot of biological diversity [14]. Estimates suggest that less than 10% of the Amazonian fungi have been formally described, despite the wide occurrence of habitats favorable to fungal development, such as organic matter, comprising rich soils, host trees, dead wood, and flooded environments [15]. The variety of ecosystems in the region—including *terra firme* (non-flooded forest), *várzea* (white-water floodplains), and *igapó* (black-water floodplains)—provides microenvironments that select for distinct fungal communities with specific adaptations and high potential for producing bioactive compounds [16,17]. In addition, the long geological history of the Amazon, whose origins date back approximately 100 million years, has favored diversification and coevolution between fungi and other organisms, resulting in unique lineages and specialized metabolic strategies. Even so, few studies have focused on the role of Amazonian fungi as sources of antimicrobials, which represents a strategic opportunity for sustainable bioprospecting and pharmaceutical innovation [18].

Although several reviews have addressed the antimicrobial potential of fungi, most have centered on cultivable groups from temperate regions, with an emphasis on ascomycetes and industrial yeasts [19,20]. Reviews specifically on basidiomycetes generally do not differentiate species from tropical regions nor integrate the ecological, taxonomic, and biochemical aspects of these organisms. Studies that mention Amazonian fungi tend to treat them secondarily or just integrate them within broader contexts of tropical biodiversity. Thus, there is a noticeable gap in the scientific literature specifically addressing the diversity of Amazonian fungi, associated study approaches like culture-based isolation, in situ surveys, or metagenomics, and the chemical potential of Amazonian basidiomycetes as producers of antimicrobial compounds. The present work aims to fill this gap by providing a comprehensive and integrated review of the topic, highlighting the Amazon as a largely unexplored frontier for the discovery of fungal metabolites of pharmaceutical interest.

### Scope and Search Strategy

The primary objective of this review is to characterize the diversity of Amazonian fungi, with emphasis on their ecology, major taxonomic groups, and the methodological approaches used to study them. We performed a structured literature review covering the period from 21 October 2005 to 21 October 2025 in Web of Science, Scopus, PubMed/MEDLINE, SciELO, and Google Scholar, using a search strategy that combined controlled terms and Boolean operators, including (“Amazon” OR “Amazonia”) AND (“fungi” OR “mycobiota”) AND (“diversity”) AND (“Basidiomycota” OR “basidiomycete”) AND (“antimicrobial” OR “bioactive metabolite” OR “secondary metabolite”). We included peer-reviewed original studies and reviews in English, Portuguese, or Spanish that (i) reported fungal diversity in Amazonian ecosystems (e.g., *terra firme*, *várzea*, *igapó*, campinarana, rivers and streams, soils, plant-associated niches, or aerobiota) using culture-based isolation, in situ macrofungal inventories, or environmental DNA/metabarcoding, and (ii) addressed basidiomycetes and/or antimicrobial activity or metabolite production.

## 2. Fungal Biodiversity in the Amazon Region

The geological and biogeographic processes that shaped the present-day Amazon Basin can be traced back to approximately 100 million years ago, which is the period of the breakup of the supercontinent Gondwana. The separation of South America from Africa, the uplift of the Andes, and the subsequent formation of the Amazon Basin provided a geological and climatic matrix highly conducive to diversification of biological groups [14]. The persistence of humid tropical forest over several dozen million years favored the coevolution of plants, animals, and microorganisms, including fungi. This long evolutionary timespan, combined with relative climate stability, created unique conditions for fungal diversification, promoting endemic lineages and specialized metabolic pathways. Nevertheless, this biodiversity remains largely unknown and under-sampled—particularly regarding fungi, whose true diversity is often opaque to traditional study methods [21].

Fungi are fundamental to the complex biological interactions within Amazonian ecosystems. As decomposers, they transform dead organic matter into recyclable nutrients, sustaining soil fertility and forest productivity. Lignocellulolytic basidiomycetes—for example, species of the genera *Ganoderma* and *Trametes*—efficiently degrade wood and leaf litter. In addition, fungi form critical symbiotic associations, such as arbuscular mycorrhizae (Glomeromycota), which enhance nutrient uptake by plants in nutrient-poor soils. Endophytic fungi colonize healthy plant tissues and can act as protectors against pathogens. In parallel, various groups of Ascomycota act as pathogens of plants and animals, regulating populations and influencing the structure of plant communities [9,10,11,12,22]. The multifunctional roles of fungi underscore their critical ecological role and their potential as environmental indicators and as sources of adaptive biomolecules [23].

**Figure 1:**
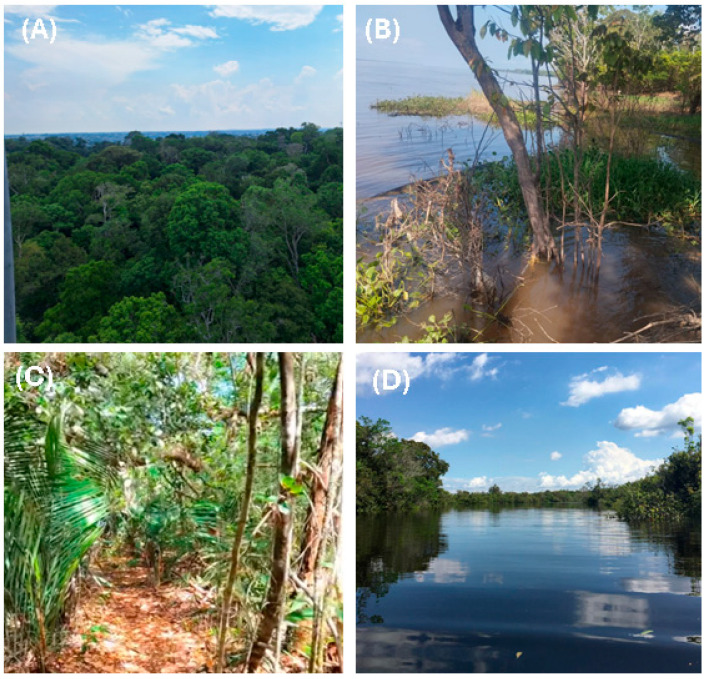
Photographic mosaic of major Amazonian landscapes (Amazonas State, Brazil): (**A**) *terra firme* (upland, non-flooded forest), Reserva Ducke (INPA), Manaus (−2.9655297, −59.9308385); (**B**) *várzea* (seasonally flooded whitewater forest), near Manaus (−3.2540665, −60.2502142); (**C**) *campinarana* (white-sand forest), Reserva da Campina (INPA), Manaus region (−2.1833330, −59.0166670); and (**D**) *igapó* (seasonally flooded blackwater forest), Rio Tarumã, Manaus (−2.9452927, −60.1105677). All photographs are from the authors’ own archive.

The Amazonian landscape comprises distinct ecosystems that directly shape the diverse composition and function of fungal communities. Examples of such ecosystems are presented in Figure 1.

*Terra firme* (unflooded upland forest), which covers about 80% of the forest, features well-drained soils and a stable hydrological environment, favoring wood-decomposing fungi and symbionts associated with large trees [24,25]. *Várzea* (whitewater floodplain forests) are seasonally flooded by sediment- and nutrient-rich whitewater, creating environments that alternate between oxygenation and anaerobiosis, thereby favoring fungi tolerant of redox fluctuations and capable of rapid substrate colonization [26,27]. *Igapó* (blackwater-flooded forests), influenced by nutrient-poor blackwater, harbor fungal communities adapted to oligotrophic and acidic conditions [27]. The microenvironmental diversity of these ecosystems promotes functional richness among fungi, ranging from specialized lignocellulolytic taxa to aquatic parasites and highly selective endophytes [17].

### 2.1. General Fungal Diversity Across Amazon Ecosystems

To frame the bioprospecting perspective of this review, we first provide an ecosystem-wide overview of Amazonian fungal diversity and the main methodological approaches used to study it. Here, we synthesize evidence from culture-based surveys, in situ inventories, and environmental DNA/metagenomic analyses, emphasizing how major Amazon ecosystems (e.g., *terra firme*, *várzea*, *igapó*, *campinarana*, and aquatic compartments) shape fungal community composition and functional roles. This section also highlights recurrent taxonomic patterns as well as key sampling and methodological biases that still constrain our understanding of the regional mycobiota. Building on this general context, Section 3 narrows the focus specifically to basidiomycete diversity and ecological roles, providing a link to the subsequent discussion on basidiomycetes and their antimicrobial potential.

#### Culture-Based Surveys

Most studies on Amazonian fungi have relied on culture-based isolation, focusing on diverse sample types such as plant tissues, soils, submerged wood, insects, and air (Table 1).

Among endophytes isolated from leaves, stems, and roots of species such as *Theobroma cacao*, *Paullinia cupana*, *Hevea brasiliensis*, and *Arrabidaea chica*, the genera *Fusarium*, *Colletotrichum*, *Trichoderma*, *Pestalotiopsis*, and *Penicillium* predominate [28,29,30,31,32,33,34,35,36,37,38]. In soils from *terra firme* and Amazonian white-sand *campinas*, *Aspergillus*, *Penicillium*, *Clonostachys*, and *Trichoderma* are particularly prominent, whereas samples of submerged wood have revealed understudied fungi such as *Xylomyces* and *Aquaticola*, with potential for producing enzymes and hydrophilic metabolites [39,40]. Fungi associated with insects—including *Acremonium*, *Cladosporium*, and *Paecilomyces*—have also been frequently isolated from culicid (mosquito) larvae and other aquatic arthropods [42,43]. Collectively, these studies revealed a broad morpho-taxonomic diversity with a predominance of Ascomycota, while also highlighting limitations regarding the representation of more fastidious or slow-growing taxa.

Culture-based methods enable the isolation of vital fungi for morphological, molecular, and functional analyses; however, this approach has significant limitations. Recovery largely depends on culture-medium conditions, temperature, pH, and incubation time. Consequently, there is a tendency towards overrepresenting easy-to-grow opportunistic fungi such as *Aspergillus* and *Penicillium* but neglecting species that are less adapted to laboratory environments. Obligate mutualists, i.e., fungi showing specialized parasitic growth behavior, and microorganisms with complex metabolic requirements often do not grow on standard media. Moreover, and for the same reason of varying growth characteristics, culture does not directly inform on the relative abundance of fungi in natural settings, which can lead to distortions in ecological interpretation [45]. The diversity observed in merely culture-based assessments is therefore only a fraction of the true diversity abundant in biological samples and should be integrated with other approaches to provide a more complete and truer overview [46].

### 2.2. In Situ Inventories of Macrofungi

In situ surveys of macrofungi conducted at multiple sites across the Amazon—including Brazil, Colombia, and Peru—have revealed considerable diversity of basidiomycetes, with a notable representation of the genera *Ganoderma*, *Trametes*, *Auricularia*, *Mycena*, *Marasmius*, *Russula*, *Amanita*, and *Boletus* (Table 2).

These studies were carried out in *terra firme*, *várzea*, *igapó*, and slope forests, using direct collection of basidiomata (fruiting bodies), morphological analysis, and samples deposited in herbaria. In addition to contributing to the taxonomy and systematics of neotropical fungi, the surveys provide information on seasonality and the relative frequency of macrofungi across different ecosystems [55,56,57]. However, there is a paucity of systematic, long-term studies, which negatively interferes with the analysis of diversity patterns, endemism, and the impacts of environmental change. Morphological identification may also be limited by the absence of complete reproductive structures or by phenotypic convergence among unrelated species.

### 2.3. Metagenomic Analyses from Environmental DNA (eDNA)

The use of next-generation sequencing (NGS) approaches for analyzing environmental DNA has substantially expanded the ability to assess fungal diversity in tropical regions such as the Amazon. Studies based on this approach are listed in Table 3.

Samples of soil, leaves, aquatic sediments, and air have revealed highly complex fungal communities, with a predominance of Ascomycota (Dothideomycetes, Sordariomycetes) and a substantial abundance of Basidiomycota (Agaricomycetes, Tremellomycetes) [56,57]. Studies conducted on observation towers such as ATTO (Amazon Tall Tower Observatory) have detected basidiospores at heights of 300 m, providing evidence for the aerial dispersal of these microorganisms [66]. In *várzea* and *igapó* sediments, a high diversity of fungi has been observed with potential roles in carbon cycling, anaerobic decomposition, and methane metabolism [64,65]. Nevertheless, these studies still face technical challenges, including the scarcity of reference genomes for tropical fungi, difficulties in assigning ecological roles to operational taxonomic units (OTUs), and susceptibility to PCR artifacts. Despite these limitations, NGS enables access to a previously invisible fraction of the Amazonian fungal diversity, including phylogenetic taxa not yet described in the scientific literature.

### 2.4. Overview of Amazonian Fungal Diversity

Each of the abovementioned approaches used to study Amazonian fungal diversity is associated with specific advantages and limitations. Culture-based methods enable functional analyses and bioprospecting for biomolecules but underestimate environmental diversity. In situ surveys capture the visible biodiversity of macrofungi but their results are season-dependent and they require high taxonomic expertise. NGS, in turn, enables high-resolution community profiling and reveals cryptic and uncultured taxa, but it still lacks methodological standardization and faces bottlenecks in taxonomic and functional annotation. Combining these methodologies in integrated studies is essential for understanding the true extent of the Amazonian fungal diversity and its ecological roles [68]. Such an integrated approach is particularly relevant when the goal of aligning ecology, taxonomy, and fungal bioprospecting is aspired [15].

**Figure 2:**
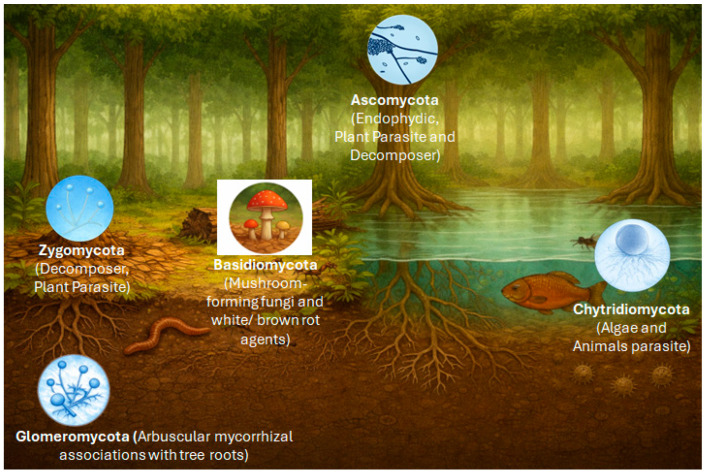
Distribution of major fungal groups across Amazonian ecosystems, integrating terrestrial and freshwater environments. The image was generated using DALL·E 3 version 3.0 (https://dalle3.ai), integrated into the ChatGPT web interface (OpenAI, San Francisco, CA, USA).

The integration of data derived from culture-based studies, in situ inventories, and metagenomic analyses provides a comprehensive overview of fungal diversity across the Amazonian landscape, as exemplarily summarized in Figure 2.

Basidiomycota dominate “*terra firme*” soils and sites of wood and leaf-litter decomposition, with an emphasis on ligninolytic decomposition [56,69,70]. Ascomycota, in turn, include numerous endophytes and plant pathogens and are frequently found in leaves, soil, and in the air. Glomeromycota are associated with the rhizosphere and are fundamental to mycorrhizal symbioses with plants in nutrient-poor soils. Zygomycota (*sensu lato;* often treated today as Mucoromycota and Zoopagomycota) occur as saprotrophs on accessible, rapidly degradable organic substrates [71,72]. Chytridiomycota are linked to aquatic environments, parasitizing algae and degrading submerged detritus. This functional and ecological diversity underscores the importance of fungi as structural and dynamic components of Amazonian ecosystems [73]. In the following, the focus will be on Basidiomycetes as potential producers of anti-microbially active biocomponents.

## 3. Basidiomycetes Diversity and Ecological Roles in the Amazonian Ecosystem

Amazonian basidiomycetes occur across a mosaic of habitats—including *terra firme*, seasonally flooded *várzea* and *igapó*, as well as aquatic–terrestrial transition zones—and comprise a major share of macrofungal diversity documented in in situ inventories. Across the surveys summarized in Table 2, the genera most repeatedly reported include *Ganoderma*, *Trametes*, *Auricularia*, *Mycena*, *Marasmius*, *Russula*, *Amanita*, *Boletus*, *Panus*, and *Lentinus*, spanning dominant wood-decay polypores, litter/understory agarics, gelatinous fungi, and occasional ectomycorrhizal lineages [17,50,51,54].

Functionally, basidiomycetes play key ecological roles as primary decomposers of lignocellulosic biomass, contributing to carbon cycling, nutrient turnover, and the formation of organic matter. Wood- and litter-associated lineages deploy oxidative and hydrolytic enzyme systems involved in lignin and cellulose depolymerization, while inhabiting substrates are rapidly colonized and strongly contested by other microbes. This ecological setting is consistent with the hypothesis that competitive interactions on resource-rich substrates can favor chemically mediated competitive strategies, including the production of antimicrobially active secondary metabolites.

**Figure 3:**
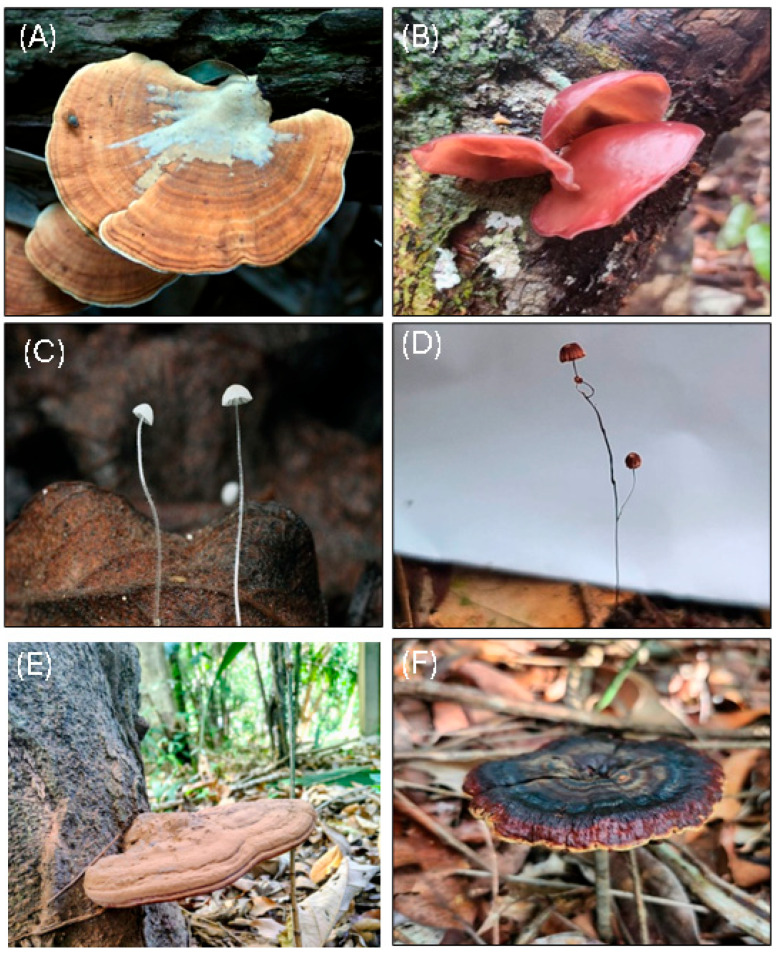
Representative Amazonian basidiomycetes encompassing major morphological and ecological groups, photographed in Reserva Ducke (INPA) (−2.9655297, −59.9308385), Manaus, Brazil, in April 2024: (**A**) *Trametes* sp. (white-rot polypore); (**B**) *Auricularia* sp. (gelatinous wood-decayer); (**C**) *Mycena* sp. (leaf-litter agaric); (**D**) *Marasmius* sp. (litter decomposer); (**E**) *Ganoderma* sp. (lignicolous poroid polypore); (**F**) *Amauroderma* sp. (tropical polypore on soil/woody substrates). All photographs are from the authors’ archive.

Representative Amazonian basidiomycetes illustrating major morphological and ecological groups are shown in Figure 3.

### 3.1. Wood Decay-Associated Polypores (Terra Firme, Várzea, Igapó)

Wood decay-associated polypores are a dominant functional component of Amazon Basidiomycota on deadwood in both upland and seasonally flooded forests. Consistent with in situ surveys (Table 2) that repeatedly reported genera include *Ganoderma* and *Trametes* (core polypore lineages), alongside Rigidoporus, Phellinus/Fomitiporia, and Hymenochaete. These taxa show extensive lignocellulolytic capacity and persistent competition on woody substrates, particularly in humid forests where decay dynamics are rapid [48].

### 3.2. Litter and Small Agarics (Leaf Litter and Understory; Primarily Terra Firme)

Leaf-litter and understory agarics contribute substantially to basidiomycete richness, especially in *terra firme* plots where microclimatic stability supports repeated fruiting. Genera most frequently associated with litter layers include *Mycena* and *Marasmius*, while recurrent litter-/wood-associated agarics such as *Panus* and *Lentinus* are also reported across Amazonian inventories. These groups colonize chemically complex substrates (litter and small woody debris) and occupy microhabitats with intense bacterial and fungal interactions, supporting their ecological relevance within the understory decomposition network [50,51].

### 3.3. Jelly Fungi and Humid Microhabitats (Decaying Logs; Shaded Strata; Flooded Forests)

Highly humid microhabitats—including decaying logs, shaded forest strata, and wetter forest compartments—support the growth of gelatinous basidiomycetes. *Auricularia* is among the most recurrently reported genera in Amazonian macrofungal surveys and is typically associated with humid, decomposing wood. Its repeated occurrence across several inventories reinforces a need for integrating gelatinous lineages into broader assessments of Amazonian basidiomycete diversity and function [17,50,51].

### 3.4. Ectomycorrhizal and Symbiotic Basidiomycetes

Although Amazonian forests are often described as predominantly arbuscular-mycorrhizal, ectomycorrhizal basidiomycetes occur in localized contexts and may remain underreported due to limited sampling and taxonomic constraints. Records of *Russula*, *Amanita*, and *Boletus/Lactarius* in Amazonian surveys highlight the importance of systematic efforts linking basidiomycete diversity to host identity, edaphic factors, and landscape context (e.g., *terra firme* vs. seasonally flooded areas) [17,49].

### 3.5. Basidiomycetous Yeasts and Airborne Basidiospores (Canopy Air Layers; Interfaces)

Beyond fruiting-body inventories, studies on environmental DNA and aerobiota indicate that basidiomycetes also occur as yeast-like lineages and as airborne basidiospores, including detections of Agaricales and Polyporales in above-canopy air layers. These data complement basidiomata-based surveys and suggest that basidiomycetes contribute to Amazonian fungal communities across multiple compartments, ranging from the canopy-associated atmosphere to aquatic–terrestrial transition zones [66,67].

### 3.6. Rationale for Prioritizing Basidiomycetes in This Review

The review’s focus is on basidiomycetes because they represent an exceptionally diverse yet comparatively underexplored reservoir of antimicrobial chemistry in comparison with traditional ascomycete-centered discovery pipelines. In Amazonian habitats—characterized by high humidity, polymer-rich plant substrates, and intense microbial competition on wood and litter—ecological pressures plausibly favor chemically mediated interactions and bioactive secondary metabolism. In parallel, advances in genomics and metabolomics increasingly reveal cryptic biosynthetic potential in basidiomycetes, supporting pathway-guided and cultural growth-based strategies (including co-culture) to access otherwise silent metabolite repertoires.

## 4. Amazonian Basidiomycetes as Sources of Novel Antimicrobials

### 4.1. Antimicrobial Resistance and the Need for New Antimicrobials

Bacterial antimicrobial resistance (AMR) is currently recognized as one of the most important threats to global public health. In 2019, approximately 4.95 million deaths were associated with AMR, with about 1.27 million directly attributable to infections caused by resistant bacteria. These numbers, taken from the Global Research on Antimicrobial Resistance (GRAM) analysis, were interpreted as linked to a decline in the efficacy of antibiotics commonly used in human and veterinary medicine [74]. In response to this situation, the World Health Organization (WHO) published a list of priority pathogens, including *Escherichia coli*, *Klebsiella pneumoniae*, *Pseudomonas aeruginosa*, *Acinetobacter baumannii*, *Staphylococcus aureus*, and *Enterococcus faecium*, many of which exhibit resistance to multiple classes of antimicrobials [75]. These microorganisms have been associated with respiratory, urinary, bloodstream, and wound infections, particularly affecting hospitalized and immunocompromised patients. The pace at which resistance emerges exceeds the rate at which new antibiotics are discovered, underscoring a need to identify innovative and effective therapeutic alternatives [76].

While antibacterial resistance is a well-known global concern, fungal infections are also a major and growing threat to human and animal health. Invasive mycoses are associated with high morbidity and mortality, especially in immunocompromised patients. Antifungal therapy remains constrained to a few substance classes, with limited options for salvage or step-down treatment. This problem has become more urgent with the rise of drug-resistant and healthcare-associated pathogens, notably *Candida auris*, which can spread in hospitals and is frequently fluconazole-resistant (e.g., ~90% of tested isolates in the U.S.) [77]. In parallel, fluconazole-resistant *Candida* species such as *C. parapsilosis*, *Candida krusei* and azole-resistant *Candida glabrata* further complicate management and stewardship [77]. Taken together, these trends underscore a need for new antifungal agents and mechanisms of action, as also indicated by global prioritization efforts focusing attention on antifungal resistance and high-impact fungal pathogens.

Against the backdrop of slow antibiotic discovery using traditional approaches, bioprospecting of natural resources has re-emerged as a promising strategy. Among organisms with the greatest biosynthetic potential, fungi have historically stood out as sources of innovative antibiotics—such as penicillin, cephalosporins, and griseofulvin—and continue to be investigated for new therapeutic classes [78,79,80]. In particular, tropical basidiomycetes are emerging as an underexplored taxon endowed with high metabolic capacity and capable of biosynthesizing compounds with complex chemical structure. Inhabiting diverse ecosystems such as *terra firme*, *várzea*, and *igapó*, these macrofungi are subjected to intense selective pressures comprising competition for substrates, extreme environmental variation, and microbial interactions that favor the production of substances with antimicrobial activity. The Amazon, which harbors one of the most extensive diversities of basidiomycetes on the planet, represents an untapped reservoir of molecules with pharmaceutical potential, reinforcing the importance of integrated studies spanning ecology, mycology, and natural products chemistry [17,27,81].

### 4.2. Antimicrobially Active Substance-Producing Basidiomycetes

In situ surveys conducted at the Brazilian Amazon and in adjacent regions in Colombia and Peru have documented a high diversity of macrofungal basidiomycetes distributed across distinct ecosystems. The genera most frequently recorded include *Ganoderma*, *Trametes*, *Auricularia*, *Mycena*, *Panus*, *Marasmius*, *Rigidoporus*, *Amauroderma*, *Polyporus*, and *Amanita* [17,82,83,84,85]. Many of these genera are recognized in scientific literature for producing bioactive compounds with antimicrobial properties. Chemical substances with antimicrobial properties have been described in basidiomycetes, with the most relevant classes including terpenes (monoterpenes, sesquiterpenes, diterpenes, and triterpenes), steroids, anthraquinones, alkaloids, quinolines, peptides, and phenolic metabolites [86,87]. Table 4 summarizes key antimicrobial compounds isolated from basidiomycetes, categorizing them by chemical class, producing species, target microorganisms, and bibliographic references.

Notable examples include terpenes (e.g., enokipodin A and grifolin), steroids, quinones, anthraquinones, alkaloids, phospholipids, and bioactive peptides. Many of these compounds exhibit high selectivity against Gram-positive bacteria such as *Staphylococcus aureus* and *Bacillus cereus*, whereas others—such as quinoline-derived alkaloids and lysophospholipids—show activity against Gram-negative pathogens like *Pseudomonas aeruginosa*, a microorganism of major clinical relevance in severely ill patients [107,108]. Notably, species such as *Ganoderma pfeifferi*, *Cortinarius mussivus*, *Gymnopus* spp., and *Ophiocordyceps sinensis* (syn. *Cordyceps sinensis*) are considered as promising sources of new antimicrobials [96,97,106,109,110]. Reports of “in vitro” activities comparable to—or even exceeding—those of conventional antibiotics, such as meropenem, underscore the largely untapped therapeutic potential of these fungi [111,112]. In addition, the table highlights some recent studies (2020—2025) exploring new metabolites, thereby expanding available research options for future drugs. These findings reinforce the importance of bioprospecting native Amazonian basidiomycetes as an alternative in times of increasing antimicrobial resistance rates.

**Figure 4:**
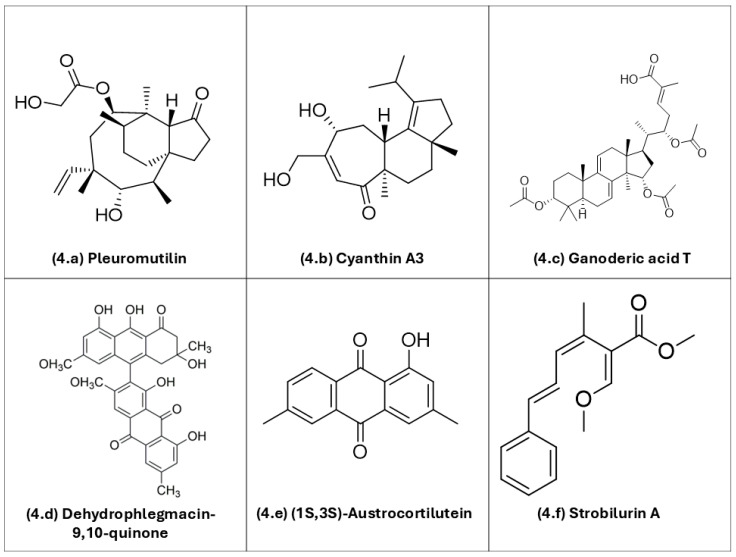
Representative chemical structures of bioactive metabolites produced by basidiomycetes, including different classes of compounds with pharmacological potential: (**4a**) pleuromutilin; (**4b**) cyanthin A3; (**4c**) ganoderic acid T; (**4d**) dehydrophlegmacin-9,10-quinone; (**4e**) (1S,3S)-austrocortilutein; (**4f**) strobilurin. ChemDraw Ultra v. 12.0 (Cambridgesoft Corp., Cambridge, MA, USA) was used to draw the chemical structures.

Figure 4 depicts the chemical structures of selected basidiomycete-derived metabolites with documented antimicrobial potential.

As detailed below, several metabolite classes—terpenes, polyketides, and quinones—and their mechanisms of action have been explored in highlighted studies that culminated in the identification of bioactive compounds from basidiomycetes:

*Sesquiterpenes and other terpenes:* Pleuromutilin (**4a**), a diterpene antibiotic originally isolated from *Clitopilus passeckerianus*, is the parent scaffold of modern pleuromutilins (e.g., lefamulin). It is active even against methicillin-resistant *Staphylococcus aureus* (MRSA) due to a different molecular mechanism of action in comparison with beta-lactam antibiotics [91,92,93]. Cyanthin A3 (**4b**), a cyathane-type diterpenoid from *Cyathus striatus*, exemplifies metabolites of basidiomycetes popularly known as bird’s-nest fungi with reported antibacterial and antifungal activities [94,95]. Ganoderic acid T (**4c**), a triterpenoid from *Ganoderma lucidum*, has shown antibacterial effects. Proposed modes of action of *Ganoderma* triterpenoids include perturbation of bacterial envelopes, although precise targets for individual ganoderic acid-based substances remain under investigation [12].

*Quinone:* Dehydrophlegmacin-9,10-quinone (**4d**), isolated from *Cortinarius mussivus*, inhibits growth and siderophore production in *Pseudomonas aeruginosa*. This observation confirms redox-active quinones as antimicrobial options [103]. (1S,3S)-austrocortilutein (**4e**), an anthraquinone from *Cortinarius basirubescens*, shows direct activity against *S. aureus*. For related *Cortinarius* metabolites (e.g., physcion, emodin), activity against *P. aeruginosa* has also been observed [97,113]. Together, these examples underscore the multifunctional potential of basidiomycete metabolites—acting on diverse cellular targets, from membranes and organelles to virulence pathways—and their potential value as candidates for new antimicrobial active drugs amid rising antimicrobial resistance rates.

*Polyketide:* Strobilurin A (**4f**), a polyketide from *Strobilurus tenacellus*, blocks mitochondrial electron transport at the Qo site of the cytochrome *bc*_1_ complex and is the progenitor of QoI-type antifungal agents [114].

### 4.3. Metabolic Pathways Involved in the Production of Anti-Microbially Active Substances of Basidiomycetes

**Figure 5:**
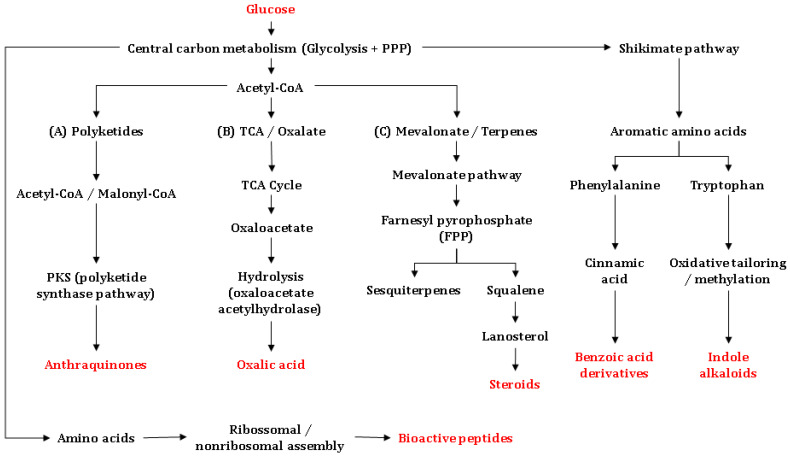
Biosynthetic origins of the main antimicrobial metabolites reported from Basidiomycetes, integrating polyketide (PKS), mevalonate (MVA), shikimate-derived aromatic, amino acid–derived alkaloid, organic-acid, and peptide (ribosomal/non-ribosomal) pathways. Microsoft^®^ PowerPoint^®^ v. 2511 (Microsoft Corp., Washington, DC, USA) was used to draw the figure.

Among the principal mechanisms underpinning the biosynthesis of antimicrobially active substances are the mevalonate pathway, which is essential for the formation of terpenes, triterpenes, and steroids, the polyketide synthase (PKS) routes, which yield anthraquinones and other quinonoid scaffolds, and non-ribosomal peptide synthetase (NRPS) pathways, which generate bioactive peptides with potent antimicrobial activities [113,115]. Additional routes include amino-acid-derived alkaloid synthesis from shikimate-pathway precursors (e.g., tryptophan/anthranilate), as well as the synthesis of polar metabolites such as oxalic acid, which is often linked to chemical defense. These pathways branch from central intermediates such as acetyl-CoA, malonyl-CoA, or tryptophan/anthranilate, and are executed by highly specific enzyme assemblies (Figure 5). In Amazonian basidiomycetes, biosynthetic diversity is further strengthened by local ecological selection pressure, which is defined by microbial competition and the biological activity of complex substrates. The latter can upregulate specialized secondary-metabolite gene clusters [116]. Details are provided in the following.

*Biosynthesis of sesquiterpenes and other terpenes:* Glucose is converted via glycolysis to acetyl-CoA, initiating the mevalonate pathway. This route produces isopentenyl pyrophosphate (IPP) and dimethylallyl pyrophosphate (DMAPP), which condense to farnesyl pyrophosphate (FPP) and geranylgeranyl pyrophosphate (GGPP). The latter ones are direct precursors of sesquiterpenes and diterpenes [117,118]. In Basidiomycetes, sesquiterpenes such as enokipodins and cubebol are synthesized from FPP via sesquiterpene synthases, whereas diterpenes such as pleuromutilin and cyathane-type metabolites derive from GGPP through diterpene cyclases. These compounds have been reported to display antibacterial and/or antifungal activity—commonly linked to membrane perturbation or defined macromolecular targets—although the precise mechanisms vary depending on the chemical scaffold [116,117,118]. From FPP, triterpene and steroid biosynthesis proceeds via squalene, which undergoes epoxidation and cyclization to lanosterol. Lanosterol is the core precursor to ganoderic acids, frequently detected in *Ganoderma* species [82,84,119,120]. These metabolites often exhibit antimicrobial effects associated with the disruption of the bacterial membrane integrity; their interference with sterol biosynthesis is primarily relevant to antifungal activity. Triterpenoid and steroid derivatives are also common in *Fomitopsis* and *Trametes*, genera widely distributed in the Amazon [11].

*Polyketide biosynthesis:* PKS pathways use malonyl-CoA as an extender unit to assemble polyketide backbones that can be modified into anthraquinones (e.g., austrocortilutein) and other quinones (e.g., dehydrophlegmacin-9,10-quinone), among additional phenolic metabolites with notable antimicrobial activity [121]. These substances are frequently effective against Gram-positive bacteria and may retain activity against multidrug-resistant isolates due to differing modes of action. Amazonian genera—including dermocyboid *Cortinarius*—are prominent producers of anthraquinones, while *Mycena* and *Gymnopilus* contribute diverse phenolic and amino-acid-derived metabolites, often in lignocellulosic substrates [103,122,123].

Biosynthesis of quinoline/quinolinone alkaloids in basidiomycetes originates from shikimate-derived precursors (e.g., anthranilate or tryptophan via the kynurenine route), followed by oxidative cyclization and further modifying steps [124,125]. Although comparatively rare in Basidiomycota, examples such as 2-aminoquinoline (*Leucopaxillus albissimus*) have been reported. Proposed antibacterial effects include interactions on DNA/topoisomerase level, but mechanisms remain incompletely resolved and may depend on specific chemical scaffolds. Distribution of such alkaloids has been recorded in soil-inhabiting saprotrophs, consistent with nutrient-rich environments [126].

Oxalic acid is a common secondary metabolite in ligninolytic basidiomycetes. Produced via oxaloacetate/glyoxylate metabolic routes, oxalic acid serves as a metal-ion chelator and exerts indirect antifungal and antibacterial effects through acidification and nutrient complexation within the growth medium. Species of *Pleurotus* and *Trametes* produce oxalic acid in relevant quantities, particularly during wood decay [127].

Basidiomycetes also synthesize bioactive peptides via two distinct biogenetic routes: ribosomal (RiPPs) and non-ribosomal (NRPs) ones. RiPPs arise from ribosomally encoded precursors that undergo extensive post-translational modification and include defensin-like peptides and omphalotins (borosin-class RiPPs). NRPS pathways assemble non-ribosomal peptides—often cyclic or lipopeptidic—that can display antibacterial, antifungal, or antiparasitic activities (e.g., peptaibol-like products such as boletusin in *Boletus* spp.). Depending on the chemical scaffold, modes of action include membrane disruption, cell-wall/membrane interference, or inhibition of protein synthesis. Comparative genomics indicate that multiple NRPS and RiPP gene clusters remain uncharacterized in Amazonian taxa, highlighting a promising frontier for the discovery of new antimicrobially active substances [128,129].

## 5. Integrative Discussion

The fungal diversity of the Amazon region is not only quantitatively remarkable but also of qualitatively strategic importance for the discovery of new bioactive metabolites. The relationship between fungal ecology and biosynthetic capacity has been increasingly recognized in the scientific literature, especially in tropical contexts where environmental complexity enforces highly specialized biochemical adaptations. Fungi inhabiting oligotrophic environments such as *igapó* (blackwater-flooded forests) or interacting with recalcitrant substrates such as submerged wood or heavily lignified foliage often produce enzymes and secondary metabolites with distinct structures and modes of action [48,81,130]. Basidiomycota, for example, stand out as saprotrophs and symbionts in *terra firme* (unflooded uplands) and *várzea* (whitewater floodplains), actively degrading lignin and recycling nutrients. This intense metabolic activity is associated with the production of compounds such as sesquiterpenes, quinones, and bioactive peptides [81,131]. The ecological architecture of the forest—marked by heterogeneous microenvironments, niches, and trophic interactions—fosters coexistence and selective pressure on fungi that produce antimicrobial substances, many of which remain unknown [132]. Consequently, Amazonian ecology acts as a functional matrix for the expression of genes related to secondary metabolism, making the forest not only a reservoir for various species but also an ecosystem that catalyzes chemical diversity [132,133].

Although the Amazon region remains under-sampled, its fungal communities display composition and distribution patterns that differ from those of other tropical forests. Compared with the Congo Basin and Southeast Asia, the Amazon harbors a high number of endemic species and a relative predominance of Basidiomycota associated with decaying wood, mycorrhizas, and leaves. Whereas tropical Africa often concentrates fungal diversity in more open, less humid ecosystems—with a predominance of Ascomycota in soil and foliage—the Amazon region’s persistently high humidity favors the abundance of macrofungi, particularly during the rainy season [134]. In tropical Asia, ectomycorrhizal genera—especially *Russula*, *Boletus*, and *Amanita*—are highly represented; these genera also occur in the Amazon region but are recorded less frequently [135]. In addition, the chemical profiles of metabolites produced by Amazonian basidiomycetes tend to show higher structural variation, reflecting a tremendous degree of metabolic specialization [136].

A deeper understanding of Amazonian fungal diversity and its biosynthetic potential requires the integration of complementary methodological approaches. Culture-based studies are fundamental for obtaining viable strains and isolates, which are required for morphological, genetic, and biochemical characterization [137,138]. However, they capture only a fraction of the total mycobiota, typically fast-growing saprotrophs. In contrast, in situ surveys on macrofungi reveal the visible diversity of basidiomata and contribute to the taxonomic and phenological record of Amazonian fungi, with notable representation of genera such as *Ganoderma*, *Auricularia*, *Mycena*, and *Marasmius* [55,139,140]. This approach, however, depends on the presence of reproductive structures and available taxonomic expertise. Metagenomics provide a powerful alternative, allowing access to the hidden diversity of uncultured fungi as well as to the functional composition of biological communities through genetic markers [141,142]. Integrated analysis of culture-based, in situ, and metagenomic datasets not only yields a more complete picture of Amazonian fungi but also helps identify potential producers of bioactive compounds and correlating their occurrence with environmental variables [141,143]. Such methodological synergy is essential in order to overcome the limitations of individual approaches and to advance the functional characterization of the region’s fungal biodiversity.

Despite recent advances, the bioprospecting of antimicrobial compounds from Amazonian basidiomycetes still faces several challenges. Most secondary metabolites described to date have been demonstrated using a limited number of culturally grown, well-studied species, whereas hundreds of Amazonian taxa remain unexplored [46,144]. Logistic barriers (access to remote areas, permitting, maintenance of cultures under tropical conditions), technical hurdles (standardization of extraction and bioassays), and financial constraints (support for fundamental and translational research) negatively interfere with progress of regional prospecting programs. The scarcity of reference genomes and of fungus-specific analytical tools for tropical fungal lineages remains a further bottleneck, which complicates the analysis of biosynthetic pathways in omics studies. Building interinstitutional collaboration networks, strain repositories, and regional databases is essential to systematize and expand knowledge on the chemical diversity of Amazonian basidiomycetes [145]. By combining tools from ecology, biotechnology, and natural products chemistry, the Amazon region can position itself as a leading frontier for the discovery of new and innovative antimicrobials [146].

## 6. Limitations and Future Research Directions

Despite increasing interest in Amazonian fungi, the available evidence remains low and fragmented. Most datasets are concentrated near accessible research hubs, while large portions of the biome and several ecosystems remain under-sampled, particularly when seasonal differences are considered. Culture-based studies tend to favor fast-growing and readily cultivable taxa, whereas DNA-based surveys may lack species-level resolution and are susceptible to sampling design, marker choice, and reference database completeness. Progress is also constrained by practical and structural limitations, including difficult access and high field logistics costs, unevenly distributed infrastructure across the region, taxonomic bottlenecks (e.g., limited availability of specialists and laboratories), and the scarcity of high-quality reference genomes and barcodes for Amazonian fungal lineages.

To overcome these gaps, future work would benefit from coordinated, surveillance-like sampling frameworks across ecosystems and seasons, standardized metadata reporting, and integrative workflows that connect taxonomy, ecology, genomics, and chemistry. A parallel priority is to strengthen regional biorepositories and curated culture collections (including long-term preservation/cryopreservation, specimen identification, and adequate data practices), enabling reproducibility and downstream bioprospecting. Priority directions include (i) the expanding of curated reference libraries and genomic resources for Amazonian fungi, (ii) the linking of bioactivity to chemical structures and biosynthetic gene clusters, (iii) validating antimicrobial activity using comparable assays and clinically relevant microbial pathogens, and (iv) advancing conservation-aware bioprospecting that respects permitting, access, and benefit-sharing requirements.

Finally, translating promising findings into usable antimicrobial substance candidates will require early attention to toxicity, stability, scalability, and mechanism-of-action studies, ideally conducted by multidisciplinary collaborations spanning natural products chemistry, microbiology, and pharmacology.

## 7. Conclusions

The Amazon Rainforest harbors fungal diversity as indicated by culture-based, in situ, and metagenomic studies. Among culture-based investigations, endophytic fungi constitute the most thoroughly studied sub-population, with a predominance of Ascomycota, particularly the genera *Colletotrichum*, *Fusarium*, and *Aspergillus*. Ecological in situ surveys—conducted mainly in *terra firme* and *várzea*—indicate a local predominance of Basidiomycota, notably the genera *Ganoderma*, *Trametes*, *Auricularia*, *Mycena*, *Marasmius*, *Russula*, *Amanita*, and *Boletus*. Metagenomic approaches reveal an even broader diversity, including phyla that are difficult to grow in culture, such as Glomeromycota and Chytridiomycota, with more than 50% of operational taxonomic units (OTUs) lacking matches to cultured biological entities.

Amazonian basidiomycetes have proven to be promising sources of antimicrobial metabolites. Genera such as *Ganoderma*, *Trametes*, *Pleurotus*, *Lentinula*, *Pycnoporus*, and *Phellinus* are frequently reported as producers of bioactive substances showing in vitro activity against clinically relevant pathogens such as *Staphylococcus aureus* and *Escherichia coli*. The principal compound classes identified include terpenes, steroids, polyketides, alkaloids, nonribosomal peptides, and oxalic acid, whose biosynthesis involves pathways such as the mevalonate (MVA), PKS, NRPS, tryptophan/shikimate, and the tricarboxylic acid (TCA) cycle.

Despite recent progress, the breadth and bioactive potential of the Amazonian mycobiota remain largely underexplored. Integrative studies—combining isolation, metagenomics, and metabolomics—are desirable to identify new species and metabolites of biotechnological interest, particularly in times of a growing demand for innovative antimicrobials due to globally rising resistance rates.

## Figures and Tables

**Table 1:** Culture-based studies on Amazon fungal diversity, organized by sample type, origin, and most frequently reported genera.

Sample Type	Sample Sources (Grouped)	Most Frequently Reported Fungal Genera and Species	References
Plant endophytes	Cacao fruits (*Theobroma cacao*); *Hevea guianensis*; *Myrcia guianensis*; *Urena lobata*; *Hevea brasiliensis* and *H. guianensis* (Peru); *Arrabidaea chica*; *Paullinia cupana*; *Hevea* spp. and *Micrandra* spp.; aquatic and riparian plants	*Fusarium*, *Colletotrichum*, *Trichoderma*, *Pestalotiopsis*, *Penicillium*, *Diaporthe*, *Lasiodiplodia*, *Daldinia*, *Purpureocillium*, *Geotrichum*	[28,29,30,31,32,33,34,35,36,37,38]
Soil	Topsoil and litter—Campina Biological Reserve, Amazonas (AM); Amazonian Dark Earth soil (*Terra Preta*)—Tailândia, Pará (PA)	*Penicillium sclerotiorum*, *Clonostachys rosea*, *Penicillium gravinicasei*, *Aspergillus*, *Sclerotium*, *Trichoderma*	[39,40]
Submerged wood	Submerged wood—Juá and Maicá Lakes, Santarém, PA; contaminated sediments—Rio Negro, AM	*Xylomyces*, *Pseudoxylomyces*, Pleosporaceae, *Fluviatispora*, Teratosphaeriaceae, *Aquaticola*, *Lielavia*	[18,41]
Insects	Culicidae larvae—AM and Rodônia (RO); larval gut of *Stenochironomus*—Ducke Reserve and Cerrado	*Acremonium*, *Aspergillus*, *Fusarium*, *Paecilomyces*, *Cladosporium*, *Penicillium*, *Trichoderma*	[42,43]
Air	Air, guano and bat body—Meu Rei Cave, Pernambuco (PE)	*Aspergillus*, *Penicillium*, *Talaromyces*, *Cladosporium*, *Rhodotorula*, *Schizophyllum*, *Rhizopus*	[44]

**Table 2:** In situ studies on macrofungal diversity in the Amazon, arranged by survey approach, study area, and representative genera.

Approach	Study Area/Habitat	Representative Genera Reported	References
In situ (checklist)	Multiple Amazonian physiognomies *terra firme várzea*, *igapó*, open fields, slope forests	*Geastrum*, *Cyathus*, *Lycoperdon*, *Phallus*, *Scleroderma*, *Tulostoma*	[47]
In situ (survey; ecological analysis)	Amazon rainforest fragment—Santarém (Pará State, Brazil)	*Cerrena*, *Trametes*, *Rigidoporus*, *Fomitiporia*, *Hymenochaete*, *Phellinus*	[48]
In situ (basidiomata survey; herbarium-based)	Oak forests and lowlands, Colombia (ColFungi catalog + HUA Herbarium)	*Agaricus*, *Amanita*, *Russula*, *Boletus*, *Termitomyces*, *Fuscoporia*, *Ganoderma*, *Trametes*	[49]
In situ (macrofungal survey; permanent plots)	Flooded forests (*várzea*) and *terra firme*—Araracuara e Amacayacu, Colombian Amazon	*Auricularia*, *Lepiota*, *Lycoperdon*, *Cordyceps*, *Pycnoporus*, *Amanita*, *Russula*, *Austroboletus*, *Boletus*, *Lactarius*	[17]
In situ (macrofungal survey)	Palmari Natural Reserve—Atalaia do Norte (Amazonas State, Brazil)	*Auricularia*, *Daedaleopsis*, *Microporus*, *Ganoderma*, *Amauroderma*, *Mycena*, *Lentinus*, *Trametes*, *Panus*	[50]
In situ (survey)	Palmari Natural Reserve—Atalaia do Norte, Alto Solimões, Amazonas (AM), Brazil)	*Mycena*, *Marasmius*, *Auricularia*, *Polyporus*, *Ganoderma*, *Trametes*, *Panus*	[51]
In situ (survey)	Palmari Natural Reserve—Atalaia do Norte, Alto Solimões, Amazonas (AM), Brazil)	*Mycena*, *Hohenbuehelia*, *Panus*, *Ganoderma*, *Auricularia*, *Trametes*, *Amauroderma*, *Rigidoporus*, *Podoscypha*	[52]
In situ (rare species record)	Porto Velho Municipal Natural Park—Rondônia (RO), Brazil	*Scleroderma anomalosporum*	[53]
In situ (macrofungal survey)	Tenente Pimenta Training Base—Humaitá, Amazonas (AM), Brazil	*Auricularia*, *Ganoderma*, *Trametes*, *Polyporus*, *Marasmius*, *Mycena*, *Panus*, *Rigidoporus*, *Amauroderma*	[54]

**Table 3:** Environmental DNA (eDNA) studies on fungal diversity in the Amazon: arranged by sample type and major taxa reported.

Sample Type	Grouped Samples/Study Sites	Major Taxa and Main Findings	References
Soil	Soil—Cuniã and Aguarongo forests (Ecuador/Brazil); deep soil and rhizosphere—Cuniã (RO); *terra firme* soil—Caxiuanã (PA); ironstone (canga) soils—Carajás (PA); soil and litter—longitudinal transect (AM, PA, RO, AC)	Ascomycota (*Postia*, Dothideomycetes, Sordariomycetes, Eurotiomycetes); Basidiomycota (*Mortierella*, *Podila*, Agaricomycetes, Tremellomycetes); Glomeromycota; *Trichoderma*	[58,59,60,61,62]
Leaves	Leaves of *Hevea brasiliensis*—Caxiuanã and Tapajós National Forests	Basidiomycetous yeasts; *Trichoderma* spp.; ectophytic parasites; endophytes and epiphytes	[63]
Aquatic sediment	Floodplain sediments—Maicá (Amazon River) and Açu (Amazon-Tapajós confluence), dry and rainy seasons	Ascomycota (predominant); Basidiomycota (Agaricomycetes, Tremellomycetes); broad microbial diversity with potential roles in biogeochemical cycling and methane metabolism	[64,65]
Air	Atmosphere—ATTO tower (300 m), Uatumã Biological Reserve (AM); above-canopy atmosphere—K34 tower, Cuieiras Reserve (AM)	Basidiospores (Agaricales, Polyporales); Pleosporales; Xylariales; Eurotiales; *Cryptococcus*; *Hygrocybe*; *Leucocoprinus*; active and passive Ascomycota and Basidiomycota	[66,67]

Abbreviations: AC, Acre; AM, Amazonas; PA, Pará; RO, Rondônia; ATTO, Amazon Tall Tower Observatory; eDNA, environmental DNA.

**Table 4:** Antimicrobial compounds produced by basidiomycetes and related macrofungi: chemical classes, representative molecules, producing taxa, and microbial targets.

Chemical Class	Representative Compounds	Producing Taxa	Potential Microbial Targets	References
Sesquiterpenes and other terpenes	Enokipodina A, Confluentina, Grifolina	*Lentinus*, *Flammulina velutipes*, *Albatrellus flettii*, *Ganoderma pfeifferi*	Gram-positive bacteria (*S. aureus*, *B. subtilis*, *B. cereus*)	[88,89,90,91,92,93,94,95]
Steroids	3,11 Dioxolanosta-8,24(Z)-dien-26-oic acid	*Jahnoporus hirtus*	Gram-positive bacteria (*B. cereus*, *E. faecalis*)	[89]
Anthraquinones	Austrocortiluteín, Fisciona, Emodina	*Cortinarius basirubencens* and other *Cortinarius* spp.	Gram-positive bacteria (*S. aureus*)	[96,97]
Benzoic acid derivatives	Coloratina A	*Xylaria intracolarata*	Gram-positive bacteria (*S. aureus*)	[98]
Oxalic acid	Oxalic acid	*Lentinus edodes*	Antibacterial activity against phytopathogenic bacteria	[99]
Peptides and proteins	Plectasina, Peptaibol boletusina, CSAP (Cordyceps sinensis antibacterial Protein)	*Cordyceps sinensis*, *Pleurotus sajor-caju*, *Pseudoplectania nigrella*, *Boletus* spp.	Gram-positive bacteria (*B. subtilis*, *S. aureus*, *Enterococcus* spp., *Streptococcus* spp.)	[100,101,102]
Quinones	Dehydrophlegmacin-9,10-quinone e derivados (1a, 1b)	*Cortinarius mussivus*	*P. aeruginosa* (growth inhibition and siderophore interference)	[103]
Amino acid derivatives/alkaloids	Pistillarin	*Mycena renati*, *Mycena zephirus*	*P. aeruginosa*	[103]
Phospholipids	LPC 18:2 (lisofosfatidilcolinas) e 13-Oxo-octadecadienoic acid	*Cortinarius caesiocanescens*	*P. aeruginosa* (in vitro activity comparable or superior to meropenem)	[103]
Unidentified phenolics	—	*Various genera* (Cortinarius, Mycena, Ramaria)	*C. albicans*, *C. glabrata*, *S. aureus*, *K. pneumoniae*, *P. aeruginosa*	[104]
Terpenes	Various metabolites (indicative of terpenes)	*Calvatia rugosa*, *Crinipellis siparunae*, *Leucocoprinus cf*. brebissonii, *Psathyrella candolleana*, *Mycena euspeirea*, *Simocybe tucumana*	*B. cereus*, *E. coli*, *P. aeruginosa*, *S. aureus*, *C. albicans*	[105,106]
Alkaloids	Gymnopalyna A	*Gymnopus* sp.	*Chromobacterium violaceum*, *Nocardia* sp., *P. aeruginosa*, *Aspergillus clavatus*, *Mucor hiemalis*	[106]

## Data Availability

New data were not created or analyzed in this study. Data sharing is not applicable to this article.

## Abbreviations

The following abbreviations are used in this manuscript:

AC	Acre
AM	Amazonas
AMR	Antimicrobial resistance
ATTO	Amazon Tall Tower Observatory
DMAPP	Dimethylallyl diphosphate
DNA	Deoxyribonucleic acid
eDNA	Environmental DNA
FAO	Food and Agriculture Organization of the United Nations
FPP	Farnesyl diphosphate
GGPP	Geranylgeranyl diphosphate
GRAM	Global Research on Antimicrobial Resistance
IPP	Isopentenyl diphosphate
MRSA	Methicillin-resistant *Staphylococcus aureus*
MVA	Mevalonate pathway
